# Distinct plasma protein profiles after long-term remission of Cushing's disease

**DOI:** 10.1210/clinem/dgag120

**Published:** 2026-04-30

**Authors:** Britte F van der Vliet, Eleonora Camilleri, Ticiana Paes, Maxim Treep, Natasja Dolezal, Robert A Cordfunke, Jan Wouter Drijfhout, Nienke R Biermasz, Mettine H A Bos, Suzanne C Cannegieter, Frits R Rosendaal, Astrid van Hylckama Vlieg, Olaf M Dekkers, Onno C Meijer, Alberto M Pereira, Leo J Hofland, L Renee Ruhaak, Frederikus A Klok, Bart J M van Vlijmen, Richard A Feelders

**Affiliations:** Department of Clinical Epidemiology, Leiden University Medical Center, 2333 ZA Leiden, the Netherlands; Department of Clinical Epidemiology, Leiden University Medical Center, 2333 ZA Leiden, the Netherlands; Department of Internal Medicine, Division of Endocrinology, Erasmus Medical Center, University Medical Center Rotterdam, 3000 CA Rotterdam, Netherlands; Department of Internal Medicine, Roger Williams Medical Center, Providence, RI 02908, USA; Department of Clinical Chemistry and Laboratory Medicine, Leiden University Medical Center, 2333 ZA Leiden, The Netherlands; Department of Immunology, Leiden University Medical Center, 2333 ZA Leiden, The Netherlands; Department of Immunology, Leiden University Medical Center, 2333 ZA Leiden, The Netherlands; Department of Immunology, Leiden University Medical Center, 2333 ZA Leiden, The Netherlands; Department of Medicine, Division of Endocrinology, and Center for Endocrine Tumors Leiden, Leiden University Medical Center, 2333 ZA Leiden, The Netherlands; Department of Medicine, Division of Thrombosis and Hemostasis, Leiden University Medical Center, 2333 ZA Leiden, The Netherlands; Einthoven Laboratory for Vascular and Regenerative Medicine, Leiden University Medical Center, 2333 ZA Leiden, The Netherlands; Department of Clinical Epidemiology, Leiden University Medical Center, 2333 ZA Leiden, the Netherlands; Department of Medicine, Division of Thrombosis and Hemostasis, Leiden University Medical Center, 2333 ZA Leiden, The Netherlands; Department of Clinical Epidemiology, Leiden University Medical Center, 2333 ZA Leiden, the Netherlands; Department of Clinical Epidemiology, Leiden University Medical Center, 2333 ZA Leiden, the Netherlands; Department of Clinical Epidemiology, Leiden University Medical Center, 2333 ZA Leiden, the Netherlands; Department of Medicine, Division of Endocrinology, and Center for Endocrine Tumors Leiden, Leiden University Medical Center, 2333 ZA Leiden, The Netherlands; Department of Medicine, Division of Endocrinology, and Center for Endocrine Tumors Leiden, Leiden University Medical Center, 2333 ZA Leiden, The Netherlands; Einthoven Laboratory for Vascular and Regenerative Medicine, Leiden University Medical Center, 2333 ZA Leiden, The Netherlands; Department of Endocrinology and Metabolism, Amsterdam University Medical Center, 1105 AZ Amsterdam, The Netherlands; Amsterdam Gastroenterology Endocrinology and Metabolism, 1105 AZ Amsterdam, The Netherlands; Department of Internal Medicine, Division of Endocrinology, Erasmus Medical Center, University Medical Center Rotterdam, 3000 CA Rotterdam, Netherlands; Department of Clinical Chemistry and Laboratory Medicine, Leiden University Medical Center, 2333 ZA Leiden, The Netherlands; Department of Medicine, Division of Thrombosis and Hemostasis, Leiden University Medical Center, 2333 ZA Leiden, The Netherlands; Department of Medicine, Division of Thrombosis and Hemostasis, Leiden University Medical Center, 2333 ZA Leiden, The Netherlands; Einthoven Laboratory for Vascular and Regenerative Medicine, Leiden University Medical Center, 2333 ZA Leiden, The Netherlands; Department of Internal Medicine, Division of Endocrinology, Erasmus Medical Center, University Medical Center Rotterdam, 3000 CA Rotterdam, Netherlands; Holman Division of Endocrinology, Diabetes and Metabolism, Department of Medicine, NewYork University Langone Medical Center, New York, NY 10016, USA

**Keywords:** Cushing's disease, mass spectrometry, plasma proteins

## Abstract

**Objective:**

Cortisol excess in Cushing's disease (CD) induces widespread plasma protein alterations, affecting various pathways including coagulation and lipid metabolism. The extent to which these abnormalities normalize during long-term remission remains unclear.

**Design:**

Cohort study investigating plasma protein profiles in CD patients before, and during long-term remission.

**Methods:**

EDTA plasma samples of 26 CD patients were collected during active disease and during long-term remission (median 4.4 years, interquartile range 3.3-5.1). Plasma protein profiles were generated using quantitative protein mass spectrometry for 159 proteins, including 21 coagulation-related proteins, 18 complement proteins, 15 transport proteins, and 14 apolipoproteins. Protein levels before and after remission were compared with 80 controls (false discovery rate-adjusted *t*-test) with similar age and sex distribution.

**Results:**

During active CD, 78 out of 159 proteins differed from controls, including 11 coagulation proteins, 10 transport proteins, and 3 apolipoproteins. Gelsolin and extracellular matrix protein-1 were most significantly changed. Following remission, 69 proteins changed significantly relative to active disease, but normalization was incomplete. Of the 78 proteins initially altered, 56 were similar to controls upon remission. After remission, 31 proteins remained different from controls, including coagulation proteins factor IX and factor XIII A chain, apolipoprotein A-II, several complement factors and extracellular matrix protein-1.

**Conclusion:**

Active CD is associated with profound alterations in plasma protein profiles across various functional domains. Long-term remission is accompanied by substantial, but incomplete normalization of plasma proteins. Sustained plasma protein abnormalities may contribute to persistent morbidities and ongoing adverse health risks after remission of CD.

Significance Statement Even after successful treatment and long-term remission, patients with Cushing's disease remain at increased risk for thrombosis and metabolic complications. The reasons for this are not well understood, and it is unclear how the plasma protein composition recovers after remission. With targeted quantitative protein mass spectrometry, this study shows that remission is accompanied by substantial, but incomplete normalization of plasma proteins. While many return to normal, 31 out of 159 measured proteins continued to be altered. These findings provide new insights into the long-term effects of Cushing's disease, and suggest that persistent protein changes may contribute to ongoing health risks. Understanding these biochemical changes could help improve patient monitoring and therapeutic management after remission.

Endogenous Cushing's syndrome (CS) is a rare endocrine disorder characterized by chronic and excessive secretion of glucocorticoids and is associated with severe morbidity and mortality (1, 2). In approximately 70% of cases, CS is caused by a pituitary adenoma that secretes excessive adrenocorticotrophic hormone, defined as Cushing's disease (CD) (3). The chronic cortisol excess in CD results in a broad spectrum of clinical symptoms like obesity, hypertension, diabetes mellitus, and neuropsychiatric complications (4). Moreover, patients with CD have an increased risk of thromboembolic events and cardiovascular disease (5-7).

Chronic cortisol excess has profound effects on plasma proteins of various functional domains. A hypercoagulable plasma state is frequently observed in CD, as evidenced by a shortened activated partial thromboplastin time and increased plasma levels of procoagulant factors and fibrinolysis inhibitors (5, 8, 9). In addition, cortisol modulates the humoral component of the immune system by downregulating pro-inflammatory cytokines and complement components, thereby contributing to immunosuppression and increased infection susceptibility in CD (10, 11). Metabolic disturbances, including dyslipidemia, are also frequently reported and characterized by elevated levels of total and low-density lipoprotein cholesterol (LDL-c) and triglycerides, and reduced high-density lipoprotein cholesterol (HDL-c) concentrations (12).

The primary treatment for CD is transsphenoidal surgery, which leads to biochemical remission in approximately 80% of patients (13, 14). However, biochemical remission does often not result in complete clinical recovery, and residual co-morbidities frequently remain (4, 15-17). While the persistence of symptoms and co-morbidities following remission is well known to clinicians, the pathophysiological mechanism has not yet been elucidated.

The impact of remission in CD on plasma protein alterations remains less well characterized, with conflicting findings reported. A study evaluating 17 patients treated with medical therapy found no improvement in coagulation and fibrinolysis markers after 12 weeks of biochemical remission (9), whereas other studies report improvements after 6 months (18) and 1 year of remission (19). Similarly, dyslipidemia was found to persist in CD patients 1 year after remission (20), while another study reported a reduction in LDL-c levels following remission (21). These contradictory results highlight the need to further investigate the biochemical consequences of CD remission.

To date, most research has focused on short-term effects of remission, with follow-up duration ranging from 6 months to 1 year. In this early phase, persistent abnormalities may reflect the effects of glucocorticoid withdrawal and delayed recovery of the hypothalamic–pituitary–adrenal axis (22). However, CD-associated comorbidities can persist for years despite biochemical remission (23) and it remains unclear whether underlying plasma protein profile alterations also persist beyond that period. Given the systemic effects of cortisol, a comprehensive evaluation of these plasma proteins may provide further insight into the long-term consequences of CD remission. Therefore, this study aims to evaluate the plasma protein profile of patients during long-term remission compared with active CD and healthy individuals using a state-of-the-art quantitative protein mass spectrometry (QPMS) approach.

## Methods

### Study population and sample collection

This cohort study included patients treated for CD at the Pituitary Center Rotterdam (Erasmus Medical Center, Rotterdam, The Netherlands). Patients were enrolled at presentation with active CD between 2014 and 2019. Diagnosis was established according to criteria outlined in current clinical practice guidelines (13). Forty-four patients were initially included, for whom clinical and biochemical examinations were conducted prior to start of treatment, and EDTA blood was collected by vena puncture according to standard procedures at Erasmus MC. Plasma was prepared by centrifugation for 10 minutes at 3000*×g*, and 500 μL aliquots were stored at −80 °C within Erasmus MC Biobank facilities. Twenty-four-hour urinary free cortisol (UFC) levels were measured on 2 separate occasions, averaged, and expressed relative to the upper limit of normal (ULN; 133 nmol/24 hours). In this initial cohort of 44 patients, treatment consisted of pituitary surgery (*n* = 39), radiotherapy (*n* = 8), and/or medical therapy (*n* = 7). Biochemical remission was confirmed by normalization of UFC and late-night salivary cortisol. During routine clinical follow-up, after patients had been in biochemical remission for at least one year, body mass index (BMI) and comorbidities were recorded and a second EDTA blood sample was obtained. This follow-up occurred between 2020 and 2022. Of the 44 included patients, 26 achieved biochemical remission and had a follow-up sample available, and were therefore included in the present analysis. For the remaining patients, no follow-up sample was collected.

The study was approved by the Medical Ethical Committee of the Erasmus MC (MEC-2013-399) and all patients provided informed consent.

EDTA plasma samples from the previously described Prevention of Thrombosis after Knee Arthroscopy trial (24) were used as controls. In this trial, blood samples were collected from individuals scheduled for knee arthroscopy within 4 hours prior to surgery. A total of 80 participants aged 25-55 years were selected from this population (median age 45 years, interquartile range [IQR]: 30-50), of whom 60 (75%) were female, to match CD patient demographics. Only samples from participants classified as American Society of Anesthesiologists class I, indicating no underlying disease, were used. Participants with diabetes or those who later developed venous thromboembolism (VTE) were excluded.

### Quantitative targeted mass spectrometry to measure 159 plasma proteins in multiplex

Plasma protein quantification was performed by multiplexed targeted liquid chromatography-mass spectrometry. In this method, plasma proteins were digested with trypsin into peptides, some of which uniquely represented the protein of interest. These peptides were separated by liquid chromatography and introduced into the mass spectrometer, where they were selected based on their mass-to-charge ratio and detected. For accurate relative quantification, synthetic stable isotope-labeled (SIL) peptides, identical in sequence to the target peptides but heavier through ^13^C or ^15^N incorporation, were added in equal amounts to each sample as internal standards to correct for variability in sample preparation and instrument response. The ratio of endogenous to SIL peptide was then used to quantify the target protein. For external calibration, values were standardized to a pooled citrate plasma calibrator included in the measurement. Full methodological details are provided in the Supplementary Methods (25), with a concise overview given below.

We applied this approach to a targeted assay quantifying 159 plasma proteins, represented by 162 peptides, selected based on detectability in previous studies (26-28) (Table S1 (25)). These proteins span diverse functional domains, including 21 coagulation-related proteins, 18 complement proteins, 15 transport-related proteins, and 14 apolipoproteins according to annotation in the Human Protein Atlas (29, 30). Sample preparation, including digestion, was automated using the BRAVO Liquid Handling System (Agilent Technologies, Santa Clara, USA). Peptides were purified through solid-phase extraction, separated using a reversed-phase UHPLC column (EclipsePlusC18 RRHD 150 × 2.1 mm, 1.8 µm particles; Agilent Technologies) and analyzed on a triple-quadrupole mass spectrometer (Agilent 6495C; Agilent Technologies) operating in multiple reaction monitoring mode. Assay performance was confirmed prior to application in study samples and demonstrated high precision. Across 5 independent runs of citrate quality control (QC) samples, coefficients of variation were below 15% for 118 proteins and below 7.5% for 97 proteins (Table S2 (25)). These thresholds served as predefined quality criteria, and citrate and EDTA QC samples measured with study samples showed comparable variation, confirming assay performance.

### Data analysis and statistics

Plasma protein levels in active CD or remission were compared with controls by calculating fold changes (fc), defined as the ratio of the mean protein level in patients to the mean level in controls. Values > 1 indicate higher and values < 1 lower abundance than in controls. For visualization in volcano plots, fc were presented as log2 fc. Statistical testing was performed with unpaired *t*-tests on individual protein values, followed by Benjamini–Hochberg false discovery rate (FDR) correction (31). Proteins with FDR-adjusted *P* value < .05 were considered different.

Protein changes between active disease and remission within the same patients were assessed by calculating fc as the ratio of mean levels in remission to mean active disease levels. Statistical testing was performed with paired *t*-tests with FDR correction. To adjust for potential confounding by remission duration, cortisol levels during active disease and BMI change, a linear mixed model with an unstructured covariance matrix and restricted maximum likelihood estimation was fitted.

We further evaluated whether protein level changes were associated with biochemical disease severity and duration of remission. Patients were stratified by baseline UFC during active disease into mild (<2 × ULN) or moderate-severe (moderate 2–5 × ULN, and severe >5 × ULN) biochemical hypercortisolism. Protein levels were compared between disease phases and controls. Associations between duration of remission (in years) and protein changes (remission vs active disease) were evaluated with linear regression.

To explore the influence of comorbidities and comedications on the protein abundance, unsupervised hierarchical clustering with Ward's method was used (32). Clustering was performed separately for active disease samples, remission samples and the change between remission and active disease, with heatmaps annotated by age, sex, BMI, prior pituitary surgery or radiotherapy, treatment strategy during follow-up, ongoing medical therapy at remission, comorbidities, and use of antihypertensive, lipid-lowering, and oral glucose-lowering medication.

Finally, proteins were grouped into functional domains based Human Protein Atlas annotations (29, 30), which describe proteins typically detectable in plasma under normal physiological conditions. The proportion of significantly altered proteins was calculated per domain.

All analyses were performed in R studio, R version 4.4.1.

## Results

### Clinical characteristics of CD patients

Among the 26 patients with CD (77% female), median UFC level during active disease was 2.7 × ULN (IQR: 1.7-4.0). Eleven patients had mild hypercortisolism (<2 × ULN) and 15 had moderate–severe hypercortisolism (>2 × ULN, 10 moderate and 5 severe). Before inclusion, 4 patients underwent pituitary surgery and one received radiotherapy; however, these treatments were unsuccessful and CD was still active at inclusion (Table 1). After inclusion, 21 patients underwent pituitary surgery (including one patient who had previous surgery). Of these, 6 received subsequent radiotherapy and 3 underwent bilateral adrenalectomy. Preoperative medical therapy was used in 20 patients and discontinued at surgery, before achieving remission. Among the 5 patients not undergoing pituitary surgery, 1 achieved remission after adrenalectomy and 4 were in remission on steroidogenesis inhibitors.

**Table 1:** Characteristics of patients with Cushing's disease during active disease and after long-term remission

	Cushing patients (*N* = 26)	
	Active disease	Remission
Female	20 (77%)	/
Age at diagnosis (y)	45 (31, 50)	/
BMI (kg/m^2^)	27.8 (26.3, 31.1)	25.9 (23.8, 28.7)
Baseline UFC (x ULN)	2.7 (1.7, 4.0)	/
Time in remission (y)	-	4.4 (3.3, 5.1)
Time between active disease and remission assessment (y)	-	5.6 (5.0, 6.5)
Surgery^*a*^	4 (15%)	24 (92%)
Radiotherapy^*a*^	1 (4%)	6 (23%)
Steroidogenesis inhibitors^*a*^	-	4 (15%)
Adrenalectomy^*a*^	-	3 (12%)
Adrenal insufficiency	-	9 (35%)
Hydrocortisone replacement therapy	-	8 (31%)
Thyroid dysfunction	7 (27%)	10 (38%)
Hypogonadism	4 (15%)	6 (23%)
Hypertension	20 (77%)	15 (58%)
Systolic BP (mmHg)	153 (130, 169)	136 (127, 148)
Diastolic BP (mmHg)	96 (88, 107)	83 (75, 90)
Antihypertensive medication use	16 (62%)	14 (54%)
Dyslipidemia	10 (38%)	11 (42%)
LDL cholesterol (mmol/L)	3.21 (2.77, 4.00)	3.40 (2.70, 3.94)
HDL cholesterol (mmol/L)	1.50 (1.10, 1.56)	1.39 (1.25, 1.58)
Lipid-lowering medication use	5 (19%)	6 (23%)
Diabetes Mellitus	7 (27%)	4 (15%)
Impaired glucose tolerance	2 (8%)	2 (8%)
Fasting glucose (mmol/L)	5.30 (4.80, 6.10)	5.05 (4.90, 5.80)
HbA1c (mmol/mol)	39 (35, 48)	39 (35, 42)
Insulin use	3 (12%)	1 (4%)
Oral hypoglycemic medication use	7 (27%)	5 (19%)

Data are presented as median (interquartile range) or number (%). Patients could have received multiple treatment strategies; therefore, treatment categories labeled with *^a^* are not mutually exclusive. Duplicate information is indicated with the / symbol while the - symbol indicates not applicable. Numbers of available observations for variables with missing data: systolic and diastolic blood pressure, *n* = 25 in remission; LDL and HDL cholesterol, *n* = 16 in active disease and *n* = 20 in remission; fasting glucose, *n* = 22 in active disease and *n* = 20 in remission, HbA1c, *n* = 20 in active disease and *n* = 20 in remission. All other characteristics were available for all 26 patients.

Abbreviations: BMI, body mass index; BP, blood pressure; LDL, low-density lipoprotein cholesterol; HbA1c, glycosylated hemoglobin; HDL, high-density lipoprotein cholesterol; UFC, urinary free cortisol; ULN, upper limit of normal; y, year.

Following remission, BMI and systolic blood pressure decreased and the prevalence of hypertension and diabetes declined, with fewer patients requiring glucose-lowering medications (Table 1). In contrast, dyslipidemia prevalence remained stable, with comparable LDL-c and HDL-c levels between active disease and remission.

Overall, the median time from active disease to confirmed remission was 0.6 years (IQR: 0.2-1.7) and the median duration of remission was 4.4 years (IQR: 3.3-5.1 years; range 1.0-7.2 years; Table 1). Median time between sampling during active disease and long-term remission was 5.7 years (IQR: 5.0-6.5).

### Alterations in plasma protein profile during active CD compared to controls

**Figure 1:**
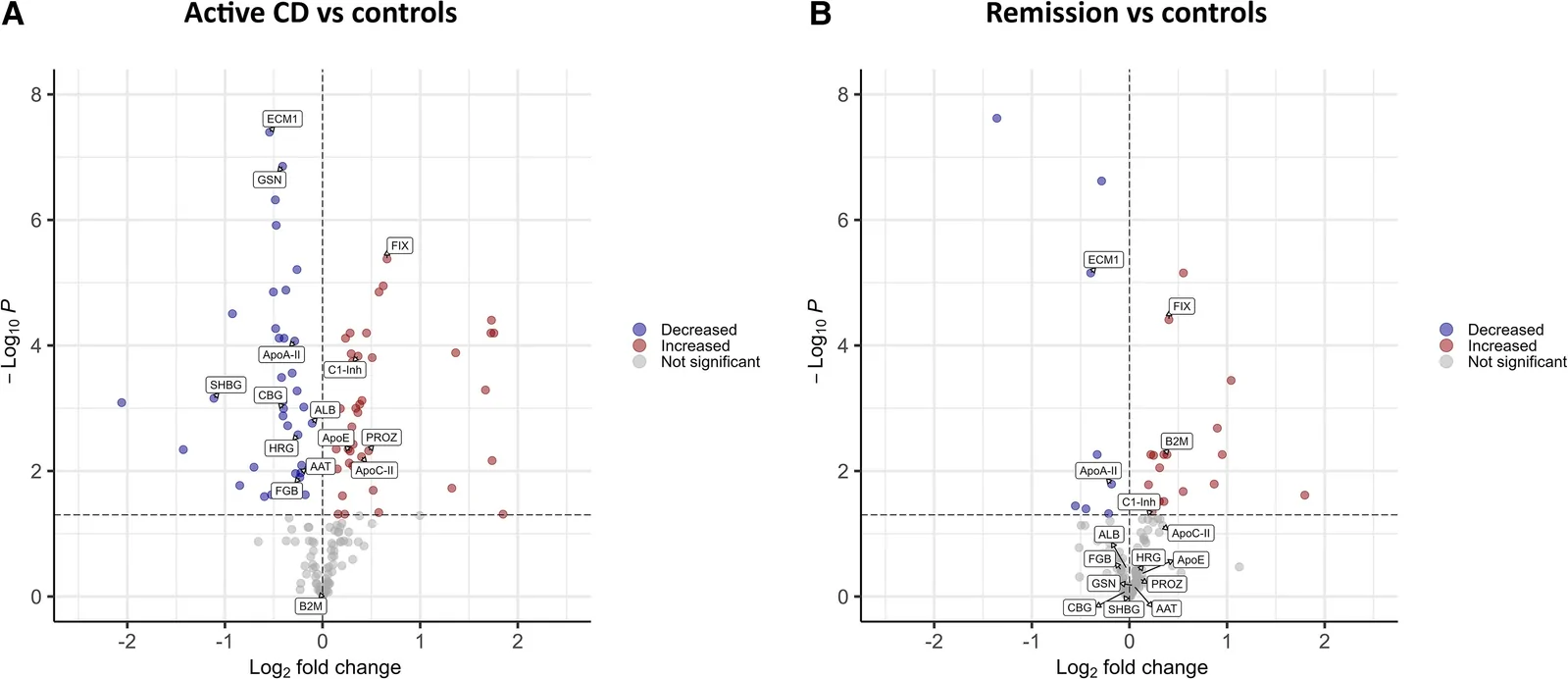
Volcano plots displaying differential plasma protein abundance in (A) active Cushing's disease and (B) remission compared to controls. The x-axis shows the log_2_ fold change in protein abundance, where positive values indicate higher levels in patients relative to controls and negative values indicate lower levels. The y-axis represents statistical significance as -log_10_(*P*-value), derived from unpaired *t*-tests with false discovery rate (FDR) correction. The vertical dotted line indicates no change (log_2_ fold change = 0), and the horizontal dotted line marks the significance threshold after FDR (*P* = .05). Proteins are color-coded according to direction of change: red for increased, blue for decreased, and gray for non-significant. Proteins of interest discussed in the text are labeled. Full results, including fold changes and *P*-values for all proteins, are provided in Table S3 (25). Abbreviations: CD, Cushing's disease; ECM1, extracellular matrix protein 1; GSN, gelsolin; FIX, coagulation factor IX; PROZ, vitamin K-dependent protein Z; C1-Inh, plasma protease C1 inhibitor; CBG, corticosteroid-binding globulin; SHBG, sex hormone-binding globulin; HRG, histidine-rich glycoprotein; AAT, alpha-1 antitrypsin; ALB, albumin; FGB, fibrinogen beta chain; APOA-II, apolipoprotein A-II; APOC-II, apolipoprotein C-II; APOE, apolipoprotein E; B2M, beta-2-microglobulin.

**Figure 2:**
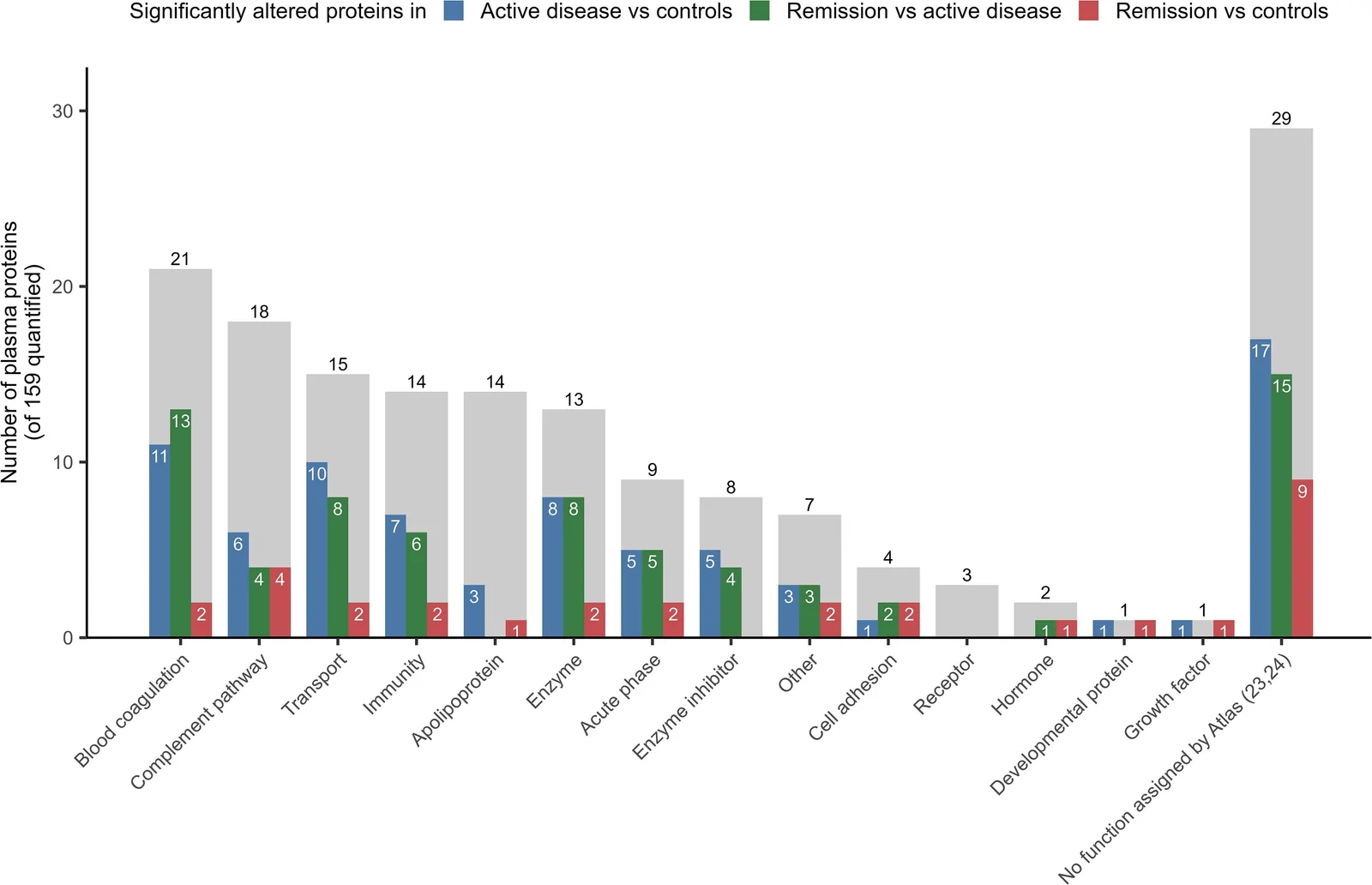
Number of significantly altered proteins per functional domain for active disease vs controls, remission vs active disease and remission vs controls. Plasma proteins (*n* = 159) were categorized according to their annotated function in the Human Protein Atlas (23, 24). Gray bars indicate the total number of plasma proteins quantified per functional domain. Colored bars indicate the number of proteins that were significantly altered (*P* < .05, false discovery rate–corrected) in active disease vs controls (blue), remission vs active disease (green) and remission vs controls (red). Numbers within colored bars denote the number of significantly altered proteins, while numbers above gray bars denote the total number of proteins measured per domain. Fold changes of individual plasma proteins are provided in Tables S3 and 4 (25).

In active CD, 78 of 159 proteins differed from controls, with 36 proteins decreased and 42 increased (Fig. 1A, Table S3 (25)), spanning multiple functional domains (Fig. 2). The most significantly altered proteins included gelsolin (GSN; fc = 0.75) and extracellular matrix protein 1 (ECM1; fc = 0.69).

Coagulation-related proteins were most affected (11 of 21 proteins, 52%). Eight were higher (including factor [F]IX: fc = 1.58; vitamin K-dependent protein Z: fc = 1.39; plasma protease C1 inhibitor: fc = 1.29), whereas 3 were lower than controls (histidine-rich glycoprotein: fc = 0.84; alpha-1-antitrypsin: fc = 0.86; fibrinogen beta chain: fc = 0.85). Transport-related proteins were also notably different from controls (10 of 15 proteins, 67%) with 7 proteins lowered, including sex-hormone-binding globulin (fc = 0.46), corticosteroid-binding globulin (CBG; fc = 0.76), and albumin (ALB; fc = 0.93).

Only 3 of 14 apolipoproteins (21%) differed, with higher levels of apolipoprotein C-II (ApoC-II; fc = 1.32) and apolipoprotein E (fc = 1.22) and lower levels of apolipoprotein A-II (ApoA-II; fc = 0.82) compared with controls.

### Changes in plasma protein profile after remission

**Figure 3:**
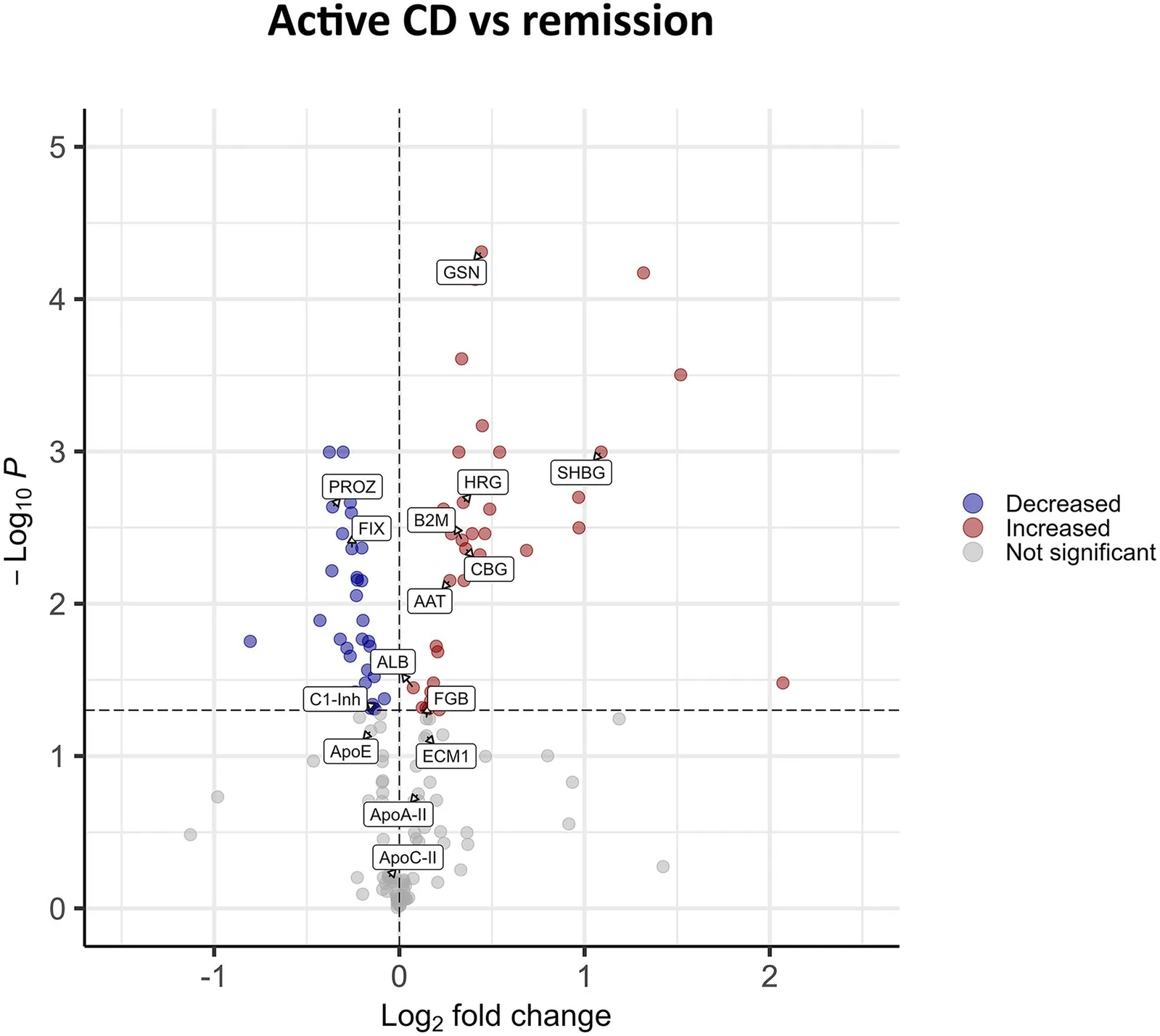
Volcano plot depicting differential plasma protein abundance between active Cushing's disease and remission within the same patient. The x-axis shows the log_2_ fold change in protein abundance, where positive values indicate higher levels during remission compared to active disease and negative values indicating lower levels during remission. The y-axis represents statistical significance as −log_10_(*P*-value), derived from paired t-tests with false discovery rate (FDR) correction. The vertical dotted line indicates no change (fold change = 1), and the horizontal dotted line marks the significance threshold after FDR (*P* = .05). Proteins are color-coded according to direction of change: red for increased, blue for decreased, and gray for non-significant. Proteins of interest discussed in the text are labeled, these are the same proteins highlighted in Fig. 1. Full results, including fold changes and *P*-values for all proteins, are provided in Table S4 (25). Abbreviations: CD, Cushing's disease; ECM1, extracellular matrix protein 1; GSN, gelsolin; FIX, coagulation factor IX; PROZ, vitamin K-dependent protein Z; C1-Inh, plasma protease C1 inhibitor; CBG, corticosteroid-binding globulin; SHBG, sex hormone-binding globulin; HRG, histidine-rich glycoprotein; AAT, alpha-1 antitrypsin; ALB, albumin; FGB, fibrinogen beta chain; APOA-II, apolipoprotein A-II; APOC-II, apolipoprotein C-II; APOE, apolipoprotein E; B2M, beta-2-microglobulin.

A total of 69 proteins changed within patients after remission compared with active disease (34 proteins decreased and 35 proteins increased as shown in Fig. 3 and Table S4 (25), with similar results after adjustment, Table S5 (25)). Of these, 52 had been altered during active disease, whereas 17 changed despite not differing from controls during active disease. Changes after remission included 13 (62%) coagulation-related proteins, 8 (53%) transport-related proteins and 8 (62%) enzymes (Fig. 2). None of the 14 apolipoproteins changed between active disease and remission.

**Figure 4:**
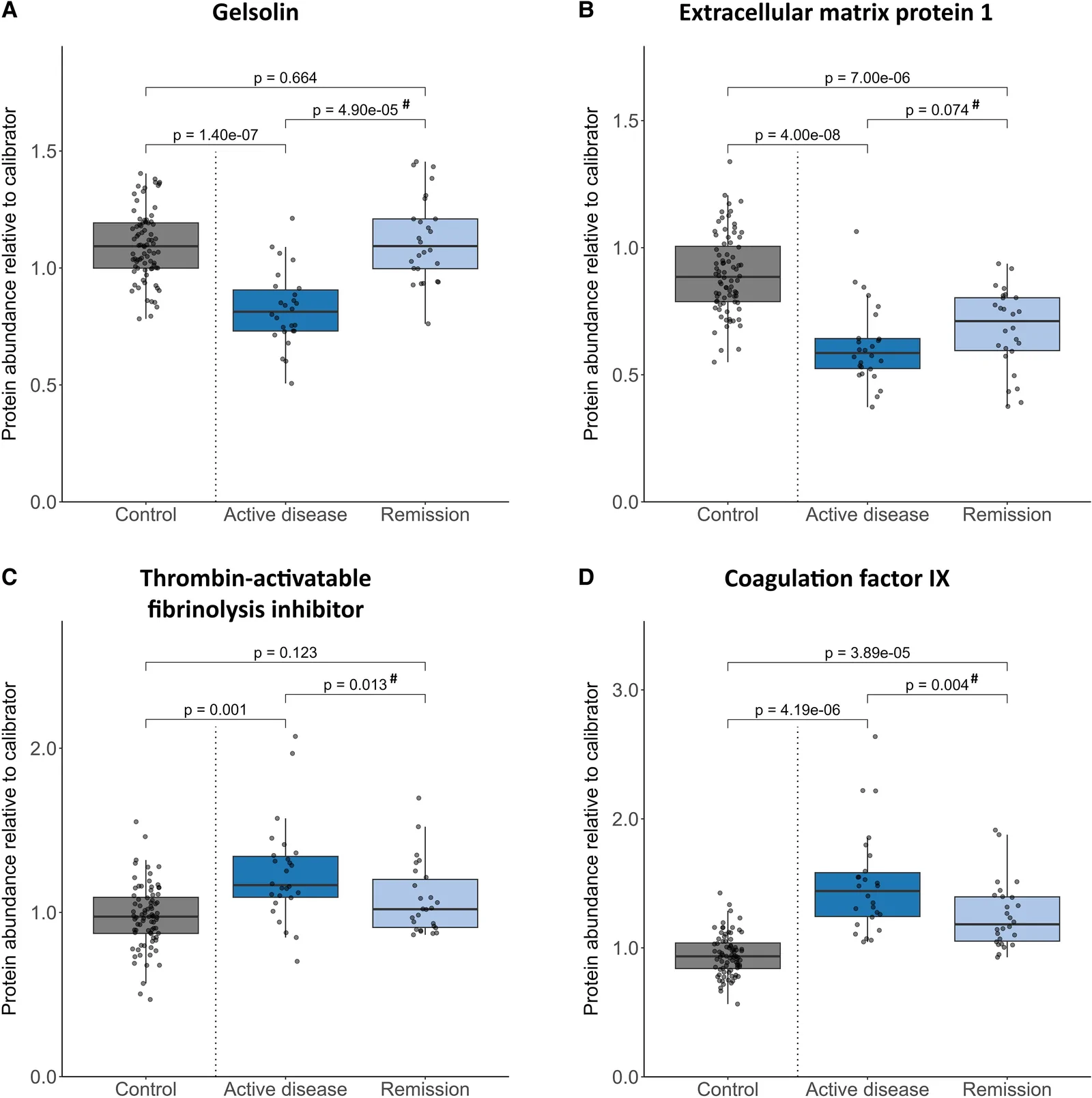
Box plots of plasma levels of (A) gelsolin, (B) extracellular matrix protein 1, (C) thrombin-activatable fibrinolysis inhibitor, and (D) coagulation factor IX in active Cushing's disease, remission, and controls. Protein levels were standardized to a pooled normal plasma calibrator. Paired t-tests with false discovery rate (FDR) correction were used for comparisons between active disease (dark blue) and remission (light blue), indicated by a # next to the *P*-value. Comparisons with the control group (gray) were assessed using unpaired t-tests with FDR correction. The dotted vertical line emphasizes the paired nature of active disease and remission samples while also indicating that control samples originate from different individuals.

Of the 78 proteins altered during active disease, 56 were no longer different from controls at remission (Fig. 1B, Table S3 (25)), with improvements across functional domains (Fig. 2). This included GSN, the most significantly altered protein in active disease, which normalized to control levels after remission (Fig. 4A). In total, 31 proteins were different from controls at remission, of which 22 were already different during active disease, suggesting persistent dysregulation. ECM1 remained among the most significantly different both in active disease and remission (Fig. 4B). In contrast, 9 proteins differed from controls only at remission, including beta-2-microglobulin, which became elevated despite being comparable with control levels during active disease (Fig. S1A (25)).

Among coagulation-related proteins, 19 of 21 were comparable to controls after remission, including 9 of the 11 previously altered in active disease, such as thrombin-activatable fibrinolysis inhibitor (Fig. 4C). In contrast, FIX remained elevated (Fig. 4D), whereas coagulation FXIIIA chain (Fig. S1B (25)) was higher only at remission. Among apolipoproteins, only ApoA-II (fc = 0.88) remained lower than controls after remission. Similarly, 13 of 15 transport-related proteins improved after remission. Only plasma serine protease inhibitor (fc = 0.39) and neutrophil gelatinase-associated lipocalin (fc = 1.46) remained different from controls. Most abnormalities persisted in the complement pathway. C2, C7, and C9 (Fig. S1C–1E (25)) remained elevated, while C1q subcomponent subunit A (Fig. S1F (25)) remained lower than in controls.

### Effect of biochemical disease severity and duration of remission on plasma protein changes

**Figure 5:**
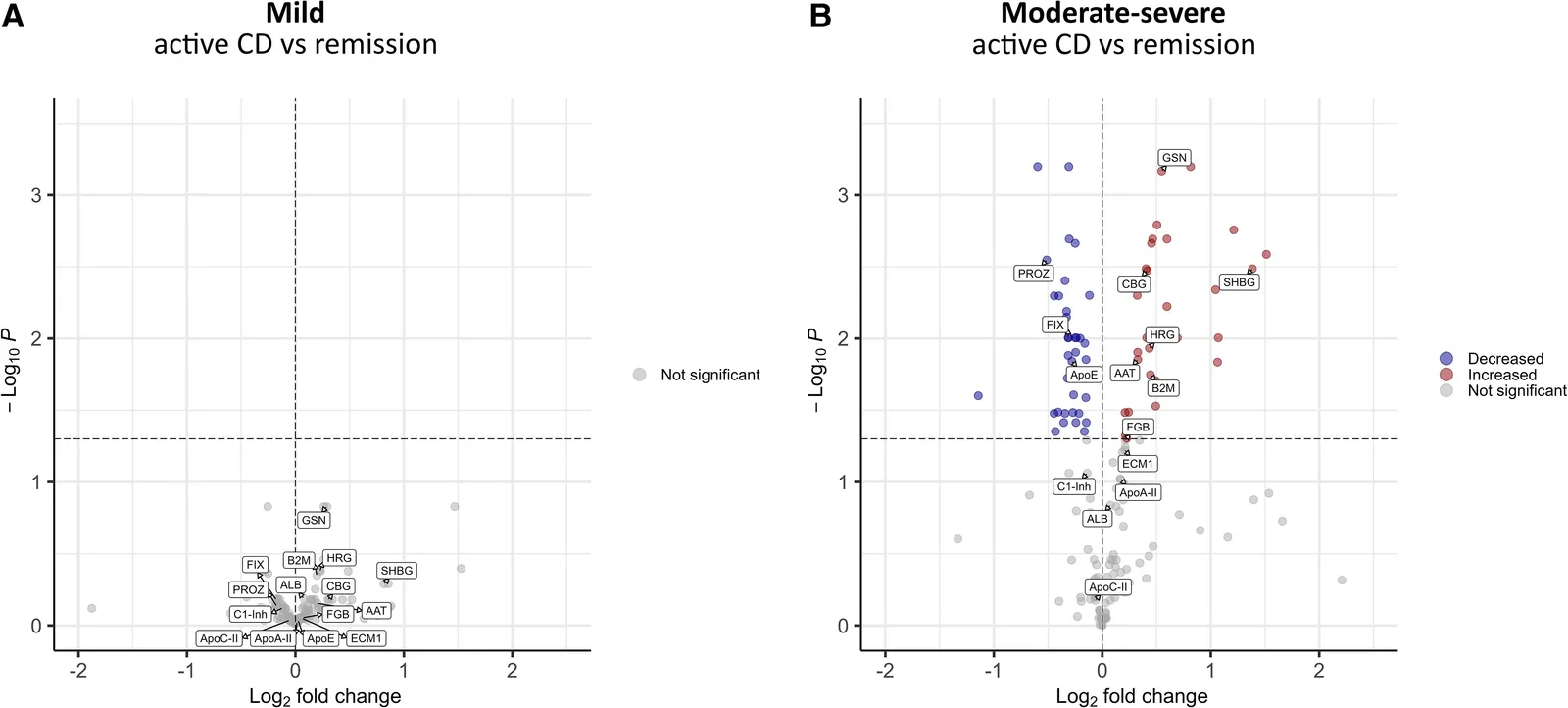
Volcano plots showing differential plasma protein abundance between active Cushing's disease and remission in patients with (A) mild and (B) moderate-to-severe hypercortisolism. The x-axis displays the log_2_ fold change in protein abundance, where positive values indicate higher levels in remission compared to active disease, and negative values indicate lower levels during remission. The y-axis represents statistical significance as –log_10_(*P*-value), based on paired t-tests with false discovery rate (FDR) correction. The vertical dashed line indicates no change (log_2_ fold change = 0), and the horizontal dashed line marks the significance threshold of *P* = .05. Proteins are color-coded by direction and significance: red for increased, blue for decreased, and gray for not significant. Proteins of interest discussed in the text are labeled, these are the same proteins highlighted in Figs. 1 and 3. Patients with mild disease (UFC <2× upper limit of normal [ULN]) included 11 individuals, while the moderate-to-severe group (UFC >2× ULN) included 15 individuals. Full results are presented in Table S8 (25). Abbreviations: CD, Cushing's disease; ECM1, extracellular matrix protein 1; GSN, gelsolin; FIX, coagulation factor IX; PROZ, vitamin K-dependent protein Z; C1-Inh, plasma protease C1 inhibitor; CBG, corticosteroid-binding globulin; SHBG, sex hormone-binding globulin; HRG, histidine-rich glycoprotein; AAT, alpha-1 antitrypsin; ALB, albumin; FGB, fibrinogen beta chain; APOA-II, apolipoprotein A-II; APOC-II, apolipoprotein C-II; APOE, apolipoprotein E; B2M, beta-2-microglobulin.

Remission duration was not significantly associated with changes in protein levels (Table S6 (25)). However, the extent of protein alterations varied by biochemical disease severity. During active disease, 30 and 66 proteins were different from controls in patients with mild and moderate–severe hypercortisolism, respectively (Table S7 (25)). No significant changes between active disease and remission were observed in the mild group (Fig. 5A, Table S8 (25)), whereas 68 proteins changed in the moderate–severe group (Fig. 5B). After remission, 28 and 17 proteins remained different from controls in the mild and moderate-severe patients, respectively (Table S9 (25)).

Unsupervised hierarchical clustering did not show clustering by comorbidities or treatment characteristics in active disease, remission, or the change between remission and active disease (Figs. S2–S4 (25)).

## Discussion

In this study, we investigated the effects of long-term biochemical remission of CD on plasma protein profile using a multiplexed QPMS approach. Our findings indicate that remission results in substantial, but incomplete normalization of the plasma protein profile. During active disease, we observed widespread alterations across functional domains. Long-term remission was accompanied by substantial shifts in protein profile, with most prominent improvements observed in transport- and coagulation-related proteins. However, abnormalities persisted, most notably within the complement system, suggesting that certain effects of chronic hypercortisolism persist despite remission. These findings highlight the systemic impact of glucocorticoid excess on plasma protein profile and suggest that associated alterations reverse only partially after long-term remission, reflecting persistent metabolic impairment. This may have implications for the long-term management of patients with CD.

The marked dysregulation of coagulation-related proteins during active CD aligns with the well-established prothrombotic phenotype in these patients. Hypercortisolism induces a hypercoagulable state through increased levels of procoagulant and antifibrinolytic proteins (9) and is associated with increased risk of VTE, both during the active phase and postoperatively (6). Our results support these findings, demonstrating moderately elevated levels of multiple coagulation-related proteins during active disease, including prothrombin, FIX and FXI. Previous studies have shown that even moderate increases in coagulation factor levels are clinically relevant; for example, every 10 IU/dL increase in FIX or FVIII levels is associated with a 7% and 11% increased risk of VTE, respectively (33). Importantly, when measured during long-term remission (median remission duration of 4.4 years [IQR 3.3-5.1]), these proteins were normalized to control levels, with the exception of FIX and FXIIIA. These findings are consistent with a previous study reporting similar reductions in procoagulant and antifibrinolytic markers one year after remission, while FIX remained elevated in some patients (19). Together, these results suggest that remission largely ameliorates the hypercoagulable state. However, VTE risk remains elevated even years after biochemical cure (7), suggesting that additional mechanisms beyond persistent coagulation abnormalities, such as endothelial dysfunction or metabolic comorbidities, contribute to sustained thrombotic risk.

We also observed marked improvements in transport proteins after remission. This broad group included various carrier proteins of vitamins, metals and steroid hormones. Of particular interest are CBG and ALB, the principal carriers of cortisol in circulation (34), which were decreased during active disease and normalized after remission. Lower levels of these proteins during active disease result in an increased proportion of unbound cortisol, thereby amplifying the biological effects of cortisol. Our findings are in line with previous findings reporting decreased CBG and elevated unbound cortisol in active CD (35). The restoration of CBG and ALB levels after remission indicates recovery of cortisol transport capacity, which may contribute to normalization of cortisol homeostasis.

CD is often characterized by decreased HDL-c, increased LDL-c and apolipoprotein alterations that may persist after remission (11). In our study, ApoC-II, associated with atherogenic lipoproteins (36), was elevated during active CD but normalized after remission, suggesting a favorable shift in lipid metabolism. Only ApoA-II, an HDL-c component (37), remained decreased after remission, whereas other HDL-associated proteins were comparable to controls. Overall, apolipoprotein changes were limited and consistent with HDL-c and LDL-c levels (Table 1), which were within normal ranges in both disease states, likely reflecting the use of lipid-lowering therapy in some patients.

Several individual proteins not previously linked to CD stood out beyond the broader domain-level changes. GSN, a key regulator of extracellular actin clearance with anti-inflammatory properties (38, 39) was markedly reduced during active disease and completely normalized after remission, suggesting strong regulation by cortisol. Low GSN levels are related to several components of atherosclerosis pathophysiology including inflammation, lipid metabolism, and thrombosis (40, 41). As GSN is predominantly produced in muscle tissue (42), its suppression may reflect glucocorticoid-induced muscle atrophy, a well-recognized feature of CD (43). In contrast, ECM1, an extracellular matrix glycoprotein involved in basement membrane organization and immune regulation (44), remained persistently decreased after remission. Although cortisol's effect on ECM1 has not been studied, glucocorticoids have been reported to affect ECM turnover (45, 46). Reduced ECM1 levels have previously been linked to fibrosis and tissue remodeling (47), indicating that its persistent decrease in CD may reflect lasting structural tissue alterations that do not resolve after remission. Together, these findings identify GSN and ECM1 as novel proteins associated with CD that may contribute to its pathophysiology and warrant further investigation.

Our results further suggest that the extent of protein profile normalization after remission may differ by biochemical disease severity during active disease. Patients with higher cortisol levels showed greater shifts in plasma protein profiles upon remission than those with milder hypercortisolism. This may reflect a dose–response relationship between degree of cortisol excess and systemic protein alterations, consistent with reports of a negative correlation between baseline UFC and persistent comorbidities after remission, with lower cortisol levels linked to diagnostic delays and prolonged glucocorticoid exposure (48). UFC values, however, do not directly capture clinical disease severity, as inter-individual differences in cortisol sensitivity and symptom presentation are well recognized (49, 50). Biochemical severity should therefore be interpreted as one aspect of disease activity rather than a comprehensive reflection of clinical impact. Overall, our findings suggest that biochemical severity is associated with the extent of protein alterations and their reversibility after remission.

Several aspects of this study warrant comment. A key strength is the availability of samples from the same patients during both active disease and long-term remission, which reduces confounding by fixed factors (51). Additionally, the relatively long follow-up allowed evaluation of sustained, long-term changes in plasma protein profile. The targeted QPMS approach provided accurate, high-throughput quantification of 159 plasma proteins with high precision. Even so, some limitations should be noted. Our sample size was relatively small, particularly for stratified analyses by biochemical disease severity, limiting statistical power. Remission samples were collected during routine clinical follow-up rather than at a standardized time interval, resulting in variability in follow-up time and remission duration between patients. Duration of cortisol excess prior to diagnosis could not be reliably quantified and may have contributed to variability in persistent protein alterations. Treatment trajectories also differed between patients, with 9 requiring sequential therapies and 4 patients remaining on steroidogenesis-inhibiting therapy at remission. Patients in surgical remission may experience different long-term clinical outcomes compared with those in remission under medical treatment (52), suggesting that treatment modality could likewise influence persistent alterations in plasma protein levels. Hydrocortisone replacement therapy and medication for cardiometabolic comorbidities may also influence circulating proteins. No distinct grouping by these factors was observed in hierarchical clustering analyses. However, more subtle effects at the level of individual proteins cannot be excluded. The available sample size precluded adequately powered subgroup analyses. Control samples were collected from individuals undergoing elective surgery, who may have had more comorbidities or inflammation than the general population, likely attenuating differences. Information on comorbidities and medication use was not available for controls due to privacy restrictions. In addition, individuals with postoperative VTE were excluded from the control group, which might have resulted in a slight underestimation of coagulation factor levels in controls. However, given the low number of such cases, the impact on our findings is likely minimal. Finally, our targeted QPMS approach inherently restricted the analysis to pre-defined, detectable proteins with concentrations above approximately 10 nM. As a result, lower abundant proteins in the low nanomolar to picomolar range, such as several endothelium-derived coagulation factors (53), were not captured despite their potential relevance. Moreover, as this approach quantifies protein concentrations rather than functional activity, complementary functional assays could help relate protein-level alterations to downstream pathological effects.

In conclusion, this study demonstrates that long-term biochemical remission of CD is associated with significant shifts in plasma protein profiles toward those observed in controls, with improvements evident across multiple physiological pathways. However, residual abnormalities persist, indicating that certain effects of CD may not be fully reversible. These findings underline the lasting systemic impact of chronic cortisol excess and highlight the need to further investigate whether persistent protein abnormalities contribute to long-term comorbidities in CD patients after remission.

## Data Availability

Some or all data sets generated during and/or analyzed during the current study are not publicly available but are available from the corresponding authors on reasonable request.

## Abbreviations

ALB: albumin
BMI: body mass index
CBG: corticosteroid-binding globulin
CD: Cushing's disease
CS: Cushing's syndrome
ECM: extracellular matrix
fc: fold changes
FDR: false discovery rate
GSN: gelsolin
HDL-c: high-density lipoprotein cholesterol
IQR: interquartile range
LDL-c: low-density lipoprotein cholesterol
QC: quality control
QPMS: quantitative protein mass spectrometry
SIL: stable isotope-labeled
UFC: urinary free cortisol
VTE: venous thromboembolism
